# Dysphagia and Dyspnea Caused by a Giant Hyalinizing Trabecular Tumor: A Case Report

**DOI:** 10.7759/cureus.79044

**Published:** 2025-02-15

**Authors:** Koji Hayashi, Yuka Nakaya, Yui Yoshida, Maho Hayashi, Rina Izumi, Asuka Suzuki, Mamiko Sato, Ei Kawahara, Yasutaka Kobayashi

**Affiliations:** 1 Department of Rehabilitation Medicine, Fukui General Hospital, Fukui, JPN; 2 Department of Internal Medicine, Fukui General Hospital, Fukui, JPN; 3 Department of Pathology, Fukui General Hospital, Fukui, JPN; 4 Graduate School of Health Science, Fukui Health Science University, Fukui, JPN

**Keywords:** acute dyspnea, dysphagia, hyalinizing trabecular tumor, thyroid gland neoplasm, thyroid neoplasm, videofluoroscopic

## Abstract

We describe the case of a 93-year-old woman with a giant thyroid neoplasm related to dyspnea and dysphagia. The patient visited our hospital for loss of appetite and dyspnea after bronchitis. Computed tomography revealed a massively enlarged thyroid gland compressing the trachea and esophagus. Cytological examination of the thyroid gland revealed features suggestive of hyalinizing trabecular tumor, a low-grade neoplasm. Videofluoroscopic swallowing examination demonstrated significant food retention and reflux in the esophagus, likely caused by upper esophageal compression. Despite recommending thyroidectomy, the patient declined surgery and received conservative and rehabilitative treatment, leading to improvement in her symptoms and discharge after 63 days.

Giant thyroid neoplasms can compress surrounding organs, leading to respiratory and swallowing difficulties. The airway's proximity to the thyroid gland makes it particularly vulnerable to compression. This case demonstrates the potential for significant respiratory and swallowing complications associated with large thyroid tumors.

## Introduction

Thyroid neoplasms encompass a diverse range of lesions that vary in their behavior and prognosis. They can be classified into benign, low-grade malignant, and malignant categories. Benign conditions, such as adenomas, are commonly encountered and typically asymptomatic. In contrast, low-grade malignant neoplasms, such as follicular thyroid carcinoma, demonstrate a more aggressive profile but often maintain a favorable prognosis [1,2]. Aggressive forms of thyroid carcinoma, including poorly differentiated thyroid cancer (approximately 5%) and anaplastic thyroid cancer (approximately 1%), are associated with poorer prognoses [2]. Notably, the overall five-year relative survival rate for thyroid cancer is estimated to be approximately 98.5%, reflecting a favorable prognosis for many patients, particularly those with well-differentiated forms like papillary thyroid carcinoma [2]. Almost all types of thyroid neoplasms can potentially present as giant thyroid tumors, which may cause symptoms due to compression of surrounding organs or tumor invasion, posing numerous challenges in surgical management [3].

A giant thyroid tumor is characterized as any thyroid tumor that weighs more than 500 grams on average and has a diameter exceeding 100 mm, with its upper edge reaching the mandibular angle and its lower edge extending to the sternum, while the posterior borders typically align with the sternocleidomastoid muscle on both sides [4]. The mechanism by which a thyroid neoplasm causes dyspnea or dysphagia may be related to several factors, including compression-related tracheomalacia [5], involvement of the recurrent laryngeal nerve [6], or compression-related esophageal insufficiency [7-10]. The most prevalent symptom in patients with obstructive cervical or substernal goiter is exertional dyspnea, occurring in 30-60% of cases [4].

Hyalinizing trabecular tumor (HTT) is a rare and controversial thyroid neoplasm predominantly observed in females in their fourth and fifth decades of life [11]. Initially regarded as a distinct entity, recent evidence indicates that it may be a variant of papillary thyroid carcinoma (PTC), as both share certain nuclear features and genetic rearrangements; however, HTT shows weaker expression of PTC markers such as cytokeratin-19 and galectin-3 [11]. Although HTT has traditionally been classified as benign, some cases have demonstrated malignant characteristics and metastasis, resulting in ongoing debates about the tumor's true nature, even as the majority behave benignly [11]. To our knowledge, there have been no prior reports of HTT compressing other organs and causing clinical symptoms, including dysphagia or dyspnea.

In this report, we describe a case of dyspnea and dysphagia resulting from a giant HTT and discuss the possible etiology of these symptoms in relation to the enlarged tumor.

## Case presentation

A 93-year-old woman with a history of diabetes mellitus, hyperlipidemia, hypertension, and myocardial infarction developed a loss of appetite and dyspnea. An enlarged thyroid gland had been noted more than a dozen years earlier, and she had observed that it had become even larger in recent years. Since around the age of 88, she had gradually lost her appetite and found it difficult to eat a full meal at once, leading her to start eating smaller portions and increasing the frequency of her meals. Her weight had also gradually decreased; it was 49.8 kg at around 88 years old but had dropped to 45.1 kg by the age of 93. Additionally, she began to experience noticeable shortness of breath during physical activity and was less willing to move over the past few years. Around the age of 92, she started having trouble swallowing her saliva, leading to saliva-soaked tissues being scattered throughout the room. She visited our hospital and was diagnosed with acute bronchitis, for which she received symptomatic treatment. Three days after the initial symptom onset, she experienced difficulty moving and returned to our hospital. Her vital signs on admission were as follows: body temperature of 37.1°C, blood pressure of 175/97 mmHg, pulse rate of 117 bpm, and oxygen saturation of 93% on room air. No hoarseness was noted. Auscultation revealed clear breath sounds; however, the patient exhibited labored respirations, with accessory muscle use observed in the shoulders. On palpation, the thyroid gland was diffusely enlarged, firm, and non-tender, without discrete palpable nodules. Blood tests revealed elevated levels of white blood cells, blood glucose, calcium, D-dimer, and C-reactive protein, along with decreased levels of albumin, creatine kinase, sodium, and chlorine (Table 1). Thyroid function tests showed free T3 at 3.27 pg/mL, free T4 at 1.58 ng/dL, thyroid-stimulating hormone (TSH) at 0.015 µIU/mL, antithyroglobulin antibody at 13.3 IU/mL (reference range: <27.9 IU/mL), thyroid-stimulating antibody at 98% (reference range: <109%), and anti-thyroid peroxidase antibody at <9.0 IU/mL. These results indicated mild inflammation likely due to the viral infection and hyperthyroidism.

**Table 1:** The result of blood tests on admission. PT: prothrombin time; INR: international normalized ratio

Inspection items	Result	Reference range
White blood cell count	9600 /μl	(3300-8600)
Red blood cell count	492×10^4^ /μl	(386-492×10^4^)
Hemoglobin	13.8 g/dl	(11.6-14.8)
Blood platelet	33.4×10^4^ /μl	(15.8-34.8)
Total protein	7.9 g/dl	(6.6-8.1)
Albumin	3.6 g/dl	(4.1-5.1)
Alkaline phosphatase	109 U/l	(38-113)
Aspartate aminotransferase	18 U/l	(13-30)
Alanine aminotransferase	10 U/l	(7-30)
Lactate dehydrogenase	222 U/l	(124-222)
Creatine kinase	28 U/l	(41-153)
γ-glutamyltransferase	14 /U/l	(9-32)
Total bilirubin	0.8 mg/dl	(0.4-1.2)
Amylase	53 U/l	(44-132)
Blood urea nitrogen	11.6 mg/dl	(8.0-20.0)
Creatinine	0.72 mg/dl	(0.46-0.79)
Cholinesterase	234 U/l	(201-421)
Sodium	135 mmol/l	(138-145)
Potassium	4.1 mmol/l	(3.6-4.8)
Chlorine	96 mmol/l	(101-108)
Glucose	192 mg/dl	(73-109)
C-reactive protein	1.76 mg/dl	(0.00-0.14)
PT	12.6 sec	(9.6–13.1)
PT-INR	1.14	(0.8-1.2)
D-dimer	4.2 μg/ml	(<0.9)

**Figure 1:**
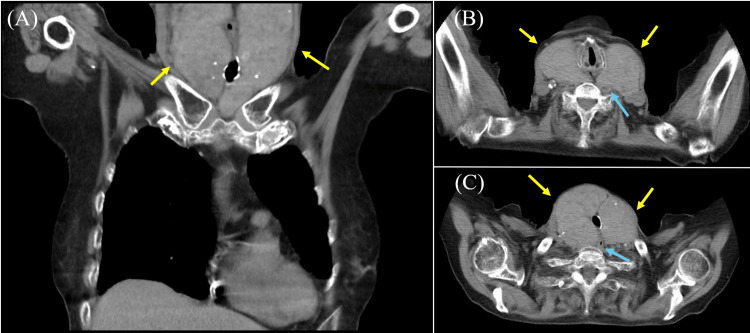
Cervical computed tomography (CT) findings. A: The coronal section of the CT scan reveals an enlarged thyroid neoplasm surrounding the airway (yellow arrows). Scattered high-density areas within the neoplasm suggest calcification. B, C: Axial sections demonstrate the enlarged thyroid neoplasm (yellow arrowhead) compressing both the airway and esophagus (blue arrowhead). The tracheal stenosis rate was calculated using the formula (normal tracheal diameter - diameter at the narrowest point)/normal tracheal diameter, resulting in a value of 53.8%. The transverse diameter of the esophagus was 6.3 mm (normal range: 20-30 mm).

**Figure 2:**
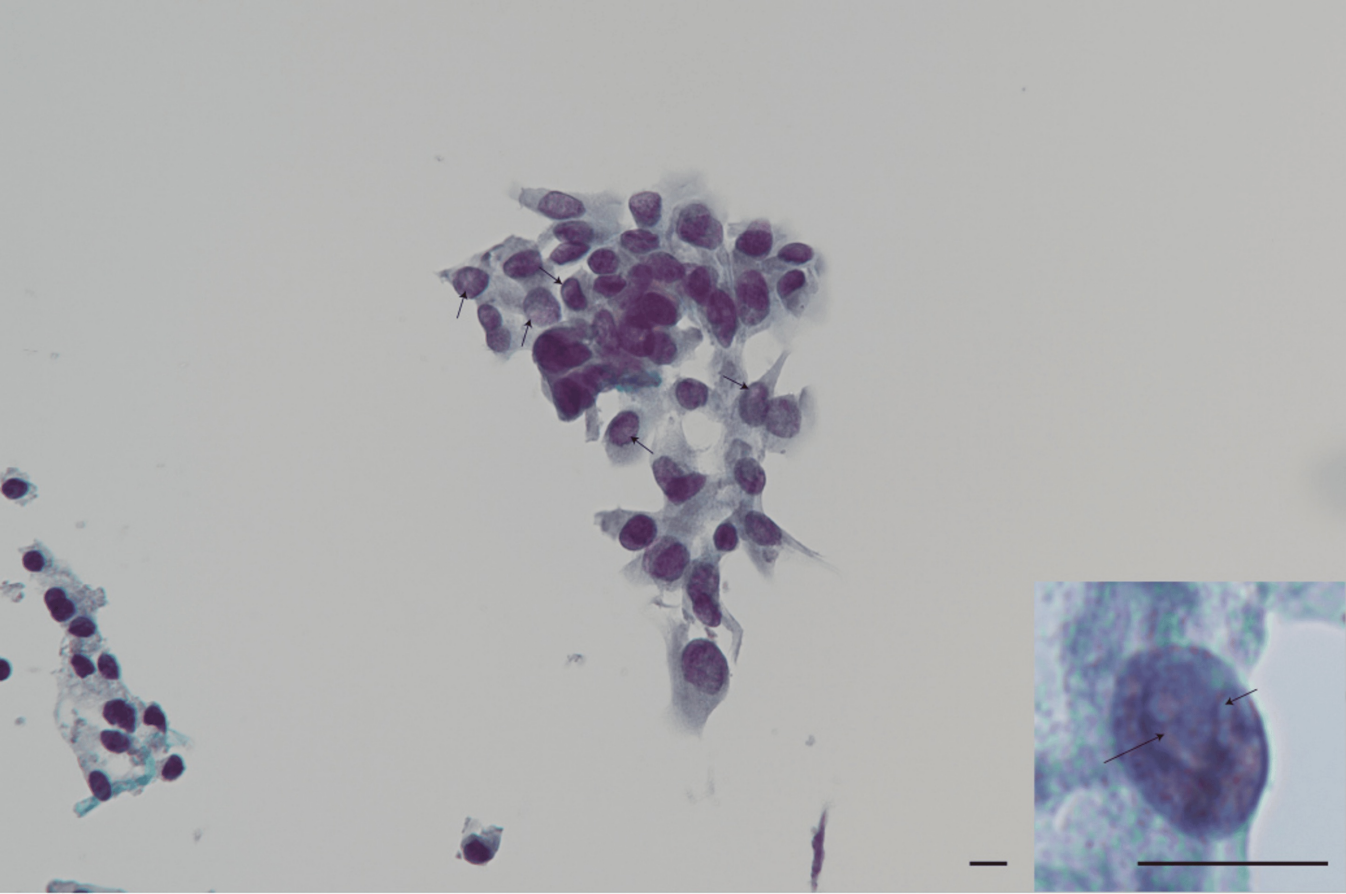
Cytologic findings of thyroid aspiration. A loosely cohesive, trabecular cluster of tumor cells is observed at the center. The nuclei of the tumor cells display large nuclei with marked anisokaryosis and irregular nuclear membranes, in contrast to the normal follicular cells on the left edge. The tumor cells also show numerous intranuclear inclusions (arrows), which suggest the possibility of papillary carcinoma or hyalinizing trabecular tumors rather than follicular tumors. The chromatin pattern of the nuclei is not ground glass but rather fine and powdery. These findings are more consistent with a hyalinizing trabecular tumor than with papillary carcinoma or follicular tumors. The inset highlights the intranuclear inclusions (arrows). Scale bar = 10 µm.

Cervical computed tomography (CT) revealed that the thyroid gland was enlarged to the extent that it compressed both the trachea and esophagus, resulting in a tracheal stenosis rate of 53.8% and a transverse diameter of the esophagus measuring 5.3 mm (Figure 1). Cytological examination of the thyroid gland revealed numerous prominent intranuclear inclusions within the cells, while intercellular cohesion was preserved (Figure 2). Based on these findings, an HTT, a low-grade neoplasm, was considered the most likely diagnosis.

A videofluoroscopic examination of swallowing (VF) revealed significant food retention and reflux in the esophagus (Video 1). Based on these findings, we hypothesized that the dysphagia was caused by a peristalsis disorder resulting from esophageal compression due to a thyroid tumor. We recommended a thyroidectomy for her, but she declined after carefully considering the risks and benefits. She received primarily conservative and rehabilitation treatment. On day 13, during the assessment at rest, her oxygen saturation (SpO2) was 99% in room air; however, she frequently coughed during meals, raising concerns about aspiration. She particularly exhibited coughing fits while drinking fluids. Based on the VF findings indicating the presence of esophageal retention and reflux, we instructed her to remain seated for at least 30 minutes after meals starting on day 15. Although her appetite was poor, with rehabilitation therapy and postural guidance, she gradually managed to increase her food intake and transitioned from a modified diet to a regular diet. However, her exertional dyspnea, including shoulder breathing, did not improve, and she continued to experience fatigue after walking just 20 meters. On day 54, she was allowed to freely consume fluids at her own discretion, and she was able to do so without any issues. With improvements noted in her respiratory symptoms and swallowing difficulties, she was discharged to a nursing facility on day 63.

**Video 1:** Videofluoroscopic examination of swallowing. This video presents a videofluoroscopic swallowing examination conducted with soft jelly. The oral and pharyngeal phases generally show good function, although some jelly residue is noted on the posterior pharyngeal wall. In the bottom right corner of the screen, the jelly can be seen passing through the esophagus; however, there is also evidence of some reflux (at 0:30 and 0:40) and retention (since 0:30) in the esophagus. The retention of food in the esophagus is presumed to indicate a decrease in peristaltic movement. In the bottom left corner, the patient is visible, and her thyroid gland appears significantly enlarged. Text in Japanese: One spoonful

## Discussion

The thyroid gland is anatomically located in front of the airways, and its enlargement can potentially compromise respiratory function. Respiratory issues, such as upper airway obstruction (UAO), are relatively common in cases of goiter and thyroid tumors. Studies report that UAO occurs in 14-31% of individuals referred to tertiary centers for goiter evaluation and in 26-60% of those referred for thyroidectomy [12]. Additionally, several cases of thyroid tumors affecting the airways have been documented, with severe exacerbations sometimes leading to acute airway obstruction [13,14]. On the other hand, asymptomatic airway obstruction has also been reported in association with thyroid enlargement [12]. We estimate that the underlying mechanisms of airway compromise in thyroid conditions include direct tracheal compression, displacement, or invasion by the enlarged thyroid tissue or tumors.

Dysphagia related to goiter or thyroid tumors has been reported to impact swallowing function. Several studies have investigated the effects of thyroid enlargement on esophageal function and swallowing mechanics. Anatomical changes have been evaluated using conventional imaging modalities. Two studies assessed the prevalence of esophageal compression and deviation in participants undergoing thyroidectomy using chest X-ray (CXR), CT, or magnetic resonance imaging (MRI) [7,8]. One study of 198 participants observed esophageal compression in 8%, esophageal deviation in 14%, or both [7]. Another study with a smaller sample size of 23 participants reported a higher prevalence of esophageal compression and/or deviation of 27% [8].

Beyond structural changes, studies have also shown the functional impact of goiters on swallowing. For instance, in participants with large goiters (>7 cm in length or >3 cm in depth on ultrasound), scintigraphically measured esophageal transit time was prolonged by two to seven seconds in 39% of participants, compared to those with smaller goiters [9,10]. Further investigation using water manometry revealed that 50% of participants with goiters exhibited esophageal motility disturbances [15]. Within that subgroup, 25% showed dysmotility of the upper esophageal sphincter, while 36% presented with a hypertonic lower esophageal sphincter; these findings were identified as potential contributors to swallowing impairment [15]. Furthermore, a detailed analysis of swallowing mechanics revealed that approximately 90% of individuals with goiters exhibited abnormalities such as elevated hyoid bone positioning, epiglottis tilting, or bolus retention in the pharynx [16].

Another significant mechanism of dysphagia in thyroid disease involves the recurrent laryngeal nerve [6]. Thyroid tumors can affect this nerve through compression, stretching, or direct invasion [17]. Recurrent laryngeal nerve damage may result in vocal cord paralysis and laryngeal dysfunction, potentially leading to swallowing disorders and increased aspiration risk [18].

These findings collectively demonstrate that goiters or thyroid tumors can cause dysphagia through multiple mechanisms, including direct structural compression, altered esophageal motility, and neurological impairment.

In our case, the patient presented with symptoms of loss of appetite and dyspnea. The clinical course was characterized by the development of symptoms such as anorexia and dyspnea during physical activity, concurrent with thyroid enlargement. This progression differed from the rapid growth and neural invasion commonly seen in undifferentiated thyroid cancer or other high-grade tumors. Cervical CT revealed significant growth of the thyroid tumor, resulting in circumferential compression of both the trachea and esophagus. Cytological examination of the tumor indicated a diagnosis most consistent with a hyalinizing trabecular tumor, a low-grade neoplasm. While hyalinizing trabecular tumors may require surgery for thyroid enlargement [19], to our knowledge, there have been no reports of hyalinizing trabecular tumors growing large enough to compress other organs and cause clinical symptoms. Although there were no abnormalities in the patient's breath sounds upon admission to the hospital, she was using accessory respiratory muscles to breathe. Given the imaging findings that indicated significant airway narrowing, we hypothesized that dyspnea could occur even with mild respiratory tract infections. VF demonstrated the thyroid tumor compressing the upper esophagus, along with evidence of esophageal phase dysfunction during swallowing, characterized by food retention and regurgitation. Notably, there was no evidence of vocal cord paralysis, suggesting that the recurrent laryngeal nerve was not involved. These findings led to the conclusion that the enlarged thyroid tumor caused direct compression of both the trachea and esophagus, resulting in dyspnea and dysphagia due to impaired esophageal peristalsis. Additionally, the compromised esophageal passage, with resultant food retention, likely contributed to the patient’s anorexia.

This case report has two limitations. First, the patient declined surgical treatment for the thyroid tumor, which prevented us from definitively establishing the precise relationship between respiratory symptoms and swallowing disorders. If the patient had undergone surgery and subsequently experienced improvement in these symptoms, it would have provided stronger support for our hypothesis regarding the tumor's impact. Second, follow-up evaluations of the VF study were not conducted, making it difficult to quantitatively assess the effectiveness of rehabilitation and conservative treatment. Without objective follow-up data, it remains uncertain to what extent conservative treatment was successful. Furthermore, while conservative management may provide a temporary solution, it may not prevent long-term complications. Having more objective follow-up data would have strengthened the presentation significantly. Further research is necessary to fully elucidate the underlying mechanisms of dyspnea and dysphagia associated with a giant thyroid neoplasm.

## Conclusions

We present a rare case of a giant HTT that compresses the trachea and esophagus in this report. HTT is not typically associated with large compressive masses, and this case contributes new clinical insights into HTT behavior. Giant thyroid neoplasms can cause respiratory and swallowing problems due to compression of surrounding organs. Since the airway is anatomically located directly behind the thyroid gland, its enlargement may easily lead to respiratory difficulties. VF findings revealed food retention and reflux in the esophageal layer, likely due to impaired esophageal peristalsis caused by compression from the thyroid neoplasm. Our case had several limitations, including the lack of surgical intervention and follow-up studies; thus, further research is needed to explore the mechanisms by which giant thyroid neoplasms cause dyspnea and dysphagia, which may contribute to the development of more effective diagnostic and therapeutic approaches, including conservative management for these challenging cases.
